# Hyperkalemia Caused by Potassium-Enriched Salt in a Hospitalized Patient

**DOI:** 10.7759/cureus.86704

**Published:** 2025-06-24

**Authors:** Masayoshi Kusunoki, Tatsuya Fujihara, Ryosuke Ishida, Yuji Yamamori

**Affiliations:** 1 Division of Emergency and Critical Care, Shimane Prefectural Central Hospital, Izumo, JPN; 2 Department of Anesthesiology, Shimane University Hospital, Izumo, JPN

**Keywords:** acute kidney injury, ckd(chronic kidney disease), hyperkalemia, hypertension, potassium-enriched salt

## Abstract

Hyperkalemia is a potentially life-threatening condition, particularly in patients with chronic kidney disease (CKD). Potassium-enriched salt substitutes are increasingly promoted as a dietary intervention for hypertension, but may pose risks in patients with impaired renal function. We report the case of an 88-year-old male with CKD who developed asymptomatic but marked hyperkalemia (7.5 mEq/L) during hospitalization for osteomyelitis. The patient had unknowingly consumed a potassium-enriched salt substitute brought in by his family to improve the taste of hospital meals. His renal function had previously declined due to septic and prerenal acute kidney injury. Following identification of the hyperkalemia, the patient was treated with glucose-insulin therapy, intravenous fluids, diuretics, and sodium polystyrene sulfonate, leading to normalization of serum potassium levels. This case highlights the importance of monitoring dietary potassium sources in hospitalized patients with CKD and underscores the need for patient and caregiver education regarding the risks of unregulated dietary supplements, especially potassium-based salt substitutes.

## Introduction

Hypertension is one of the major risk factors for cardiovascular disease, and salt restriction plays an important role in managing blood pressure in patients with hypertension [1]. In 2013, the World Health Organization recommended a daily sodium intake of less than 2 g, a guideline that is especially critical for hypertensive patients in Asian countries, where salt consumption is relatively high and often exceeds recommended levels [2,3]. Approximately 80% of patients with chronic kidney disease (CKD) have hypertension; interventions targeting hypertension are believed to reduce albuminuria and cardiovascular risk [4]. Recently, potassium-enriched salt has gained attention as a strategy for salt restriction in hypertensive patients [1]. However, its use may increase the risk of hyperkalemia in patients with CKD. Here, we present the case of a hospitalized septic patient with CKD who developed hyperkalemia following the ingestion of a potassium-enriched salt.

## Case presentation

An 88-year-old male presented to our emergency department with a one-day history of fever. He had a history of CKD and was regularly receiving treatment at another hospital. His medical history included diabetes mellitus, hypertension, hyperuricemia, and chronic hepatitis C. Regular medications included febuxostat 20 mg/day and amlodipine 2.5 mg/day. Physical examination was unremarkable except for swelling and pain in the left middle finger. Laboratory testing revealed renal dysfunction, with a blood urea nitrogen (BUN) of 53.4 mg/dL and creatinine (Cre) of 3.77 mg/dL. The patient’s initial potassium level was 5.3 mEq/L (Table 1).

**Table 1:** Laboratory findings during hospitalization Cre, creatinine; BUN, blood urea nitrogen; K, potassium

Test	Reference Range	Day 1	Day 4	Day 5	Day 7	Day 12	Day 15	Day 19	Day 26	Day 34	Day 35	Day 36	Day 37	Day 38	Day 39	Day 40
K (mEq/L)	3.6-4.9	5.3	4.7	4.5	4.6	4.7	5.2	5.2	5.2	7.5	7.2	6.7	5.7	5.6	4.8	4.5
BUN (mg/dL)	8.0-22.0	53.4	62.5	55.3	43.7	47.2	42.2	40.1	31.2	30.6	30.0	-	-	-	-	32.0
Cre (mg/dL)	0.60-1.10	3.77	4.55	4.10	3.52	2.96	2.90	3.08	2.84	2.88	2.85	-	-	-	-	2.79

According to the most recent laboratory results from four years prior, his baseline renal function included a BUN of 16.8 mg/dL, Cre of 1.50 mg/dL, and estimated glomerular filtration rate (eGFR) of 34.4 mL/minute/1.73 m², consistent with CKD stage G3b. Venous blood gas analysis on arrival showed the following: pH 7.353, PCO₂ 28.4 mmHg, PO₂ 37.8 mmHg, HCO₃- 15.4 mmol/L, base excess -8.7 mmol/L, and lactate 3.2 mmol/L. Urinalysis showed proteinuria and mild hematuria but was otherwise unremarkable (Table 2).

**Table 2:** Urinalysis during hospitalization HPF, high-power field; WF, whole field

Test	Reference Range	Day 1	7
Specific gravity	1.006-1.022	1.014	1.010
pH	4.8-7.5	5.5	6.5
Protein	-	3+	2+
Glucose	-	-	-
Urobilinogen (mg/dL)	0.2-1.0	0.1	0.1
Ketone body	-	-	-
Occult blood	-	3+	1+
Bilirubin	-	-	-
Nitrites	-	-	-
Red blood cells (RBC)/HPF	<5	10-19	1-4
White blood cells (WBC)/HPF	<5	1-4	1-4
Bacteria	-	1+	2+
Granular casts/WF	0-1	5-9	1-4
RBC shape	-	Mixed	-

The patient was admitted for osteomyelitis of the left middle finger, which was later confirmed by MRI imaging during hospitalization, and antibiotic therapy (ceftriaxone 2 g/day) was initiated. On the second day of hospitalization, *Streptococcus dysgalactiae* was identified in the blood culture. On the 21st day, antibiotics were switched to oral amoxicillin 500 mg twice daily, with a plan for six weeks of continued therapy. In summary, the patient’s clinical course involved *S. dysgalactiae* bacteremia and osteomyelitis, complicated by the development of acute kidney injury (AKI). There were no signs of a postrenal cause for the AKI. Given the improvement in renal function following antibiotic therapy, the patient was diagnosed with septic AKI combined with prerenal AKI secondary to bacteremia. On the 34th day of hospitalization, a blood test revealed significant hyperkalemia, with a potassium level of 7.5 mEq/L (Table 1). The blood sample was evaluated multiple times and confirmed to have no signs of hemolysis, which could cause pseudo-hyperkalemia. Despite this significant hyperkalemia, the patient remained asymptomatic, with no electrocardiographic changes, bradycardia, or hemodynamic instability. An interview revealed that the patient had received potassium-enriched salt containing 27.3% (27,300 mg/100 g) of potassium as a gift from his family during hospitalization. He reported he frequently added it to his meals because he found the hospital-provided food insufficiently salty. He had not regularly used potassium-enriched salt prior to admission; it had been newly brought in by his family solely for seasonings. Following treatment with glucose-insulin therapy, intravenous saline, loop diuretics, and sodium polystyrene sulfonate, the patient’s potassium levels rapidly decreased and normalized within a few days. He was advised to discontinue the use of potassium-enriched salt. The patient was discharged on the 40th day of hospitalization.

## Discussion

The use of potassium-enriched salt reduces sodium intake compared to regular table salt, as it substitutes sodium chloride with potassium chloride (comprising 25-67% potassium chloride), offering a convenient method for salt restriction [5]. Potassium-enriched salt is widely used globally. Its use is particularly promoted in China through public campaigns [6,7], and it has gained increasing attention in East Asia, where salt consumption is relatively high [8]. Recent studies have suggested the efficacy of potassium-enriched salts in lowering blood pressure and reducing cardiovascular disease-related mortality. One meta-analysis demonstrated reductions in systolic and diastolic blood pressure by 4.9 mmHg and 1.5 mmHg, respectively [9]. Furthermore, a randomized trial found that long-term use of potassium-enriched salt significantly reduced cardiovascular mortality (age-adjusted hazard ratio: 0.59; 95% confidence interval: 0.37, 0.95) [10]. While its antihypertensive and cardiovascular benefits are promising, the safety of potassium-enriched salt in patients with CKD remains uncertain. A modeling study predicted that replacing regular salt with potassium-enriched salt could prevent 461,000 deaths from cardiovascular disease but may cause an additional 11,000 deaths from hyperkalemia in patients with CKD [5]. The potassium-enriched salt used in the study contained 20-30% potassium chloride; similarly, potassium-enriched salt sold in Japan contains 27.3%, raising concerns about its safety in Japanese patients with CKD.

In the present case, we encountered a patient with CKD who consumed potassium-enriched salt during hospitalization, leading to the development of hyperkalemia. The antibiotics used in the present case (amoxicillin and ceftriaxone) do not contain potassium as the active ingredients. Therefore, we believe the influence of antibiotics as a cause of hyperkalemia in this patient is unlikely. A retrospective cohort study found that hyperkalemia is common among hospitalized patients, with a prevalence of 8.9% and a mean age of 64.7 years; patients with kidney failure on hemodialysis were at particularly high risk for severe cases [11]. Although dialysis was not required in this case, the patient developed hyperkalemia during hospitalization due to the unexpected intake of a dietary supplement (potassium-enriched salt). Although a uniform restriction of fruits and vegetables is not recommended according to the current CKD guideline, it is advised to assess for excessive intake of highly processed foods, meats, dairy products, juices, and salt substitutes [12]. However, neither the patient nor his family had received adequate education regarding this issue. This case highlights the risk of hyperkalemia associated with potassium-enriched salt in patients with CKD and underscores the need for physicians to educate such patients about this risk. It also emphasizes that, in hospitalized CKD patients, strict monitoring of dietary intake and careful management of brought-in foods and supplements are essential. Additionally, educating patients and their families about easily accessible items, such as potassium-enriched salt, is crucial. To prevent similar incidents, more thorough screening of outside food and supplements brought into hospitals may be necessary. Furthermore, incorporating discussions about high-risk supplements during outpatient visits may help prevent inappropriate consumption. Efforts should also be made to strengthen admission protocols and enhance communication between healthcare providers and families to prevent the unsupervised introduction of high potassium foods. Moving forward, in the care of hospitalized patients with CKD, it will be critical to establish systems that prevent the introduction of high-potassium foods and reinforce patient and family education. This may contribute to safer nutritional management. These efforts are especially important in countries such as Japan and other Asian nations, where potassium-enriched salt is easily accessible without a prescription. To reduce the risk of similar adverse events, specific interprofessional strategies may include involving nutritionists and pharmacists in regular dietary assessments and medication/supplement reviews, implementing patient and family education programs that include counseling on potassium management, and establishing standardized screening protocols for early detection of potassium imbalances in high-risk patients.

## Conclusions

Potassium-enriched salt offers benefits in terms of sodium restriction and cardiovascular risk reduction, but it also poses a risk of hyperkalemia in patients with CKD. As this risk is not widely recognized, it is essential to educate both patients and their families. In hospitalized patients, especially those with CKD, it is important to strictly manage and monitor the use of brought-in foods and supplements.
